# Inhibition of protein kinase D and its substrate phosphatidylinositol-4 kinase III beta blocks common human coronavirus replication

**DOI:** 10.1128/spectrum.01501-24

**Published:** 2024-11-14

**Authors:** Huijuan Han, Huan Liu, Rebbeca Steiner, Zhijun Zhao, Zheng-Gen Jin

**Affiliations:** 1 Aab Cardiovascular Research Institute, Department of Medicine, University of Rochester School of Medicine and Dentistry, Rochester, New York, USA; 2 Department of Medical Laboratory, School of Clinical Medicine, Ningxia Medical University; Ningxia Key Laboratory of Clinical and Pathogenic Microbiology, General Hospital of Ningxia Medical University, Yinchuan, China; 3 State Key Laboratory of Experimental Hematology, National Clinical Research Center for Blood Diseases, Institute of Hematology & Blood Diseases Hospital, Chinese Academy of Medical Sciences & Peking Union Medical College, Tianjin, China; Shandong First Medical University, Jinan, Shandong, China

**Keywords:** protein kinase D, phosphatidylinositol-4 kinase IIIbeta, common human coronavirus, replication, trans-Golgi network

## Abstract

Coronavirus disease 2019 (COVID-19) is caused by the infection of a coronavirus, named as severe acute respiratory syndrome coronavirus 2 (SARS-CoV-2). Coronaviruses can be replicated in the infected host cells. Coronavirus replication involves various steps, including membrane fusion, peri-nuclear particle formation, and matrix vesicle transport to the cell membrane *via* the endoplasmic reticulum–Golgi-lysosome route. Recent studies have suggested that protein kinase D (PKD) plays a crucial role in regulation of vesicle formation and trafficking in the trans-Golgi network (TGN). Thus, we hypothesize that inhibiting PKD and its associated pathway could be an effective strategy to limit viral replication. Here, we report that molecular and pharmacological inhibition of PKD and its substrate phosphatidylinositol-4 kinase III beta (PI4KIIIβ) significantly diminishes the replication of common human coronaviruses. Specifically, we found that the PKD-silencing siRNA and the PKD inhibitor CRT0066101 have broad-spectrum antiviral activity against HCoV-OC43, HCoV-NL63, and HCoV-229E in cultured cells. Mechanistic studies revealed that the deactivation of PKD reduced the activation of PI4KIIIβ, thereby blocking the transport of viral particles in the host cells. Furthermore, the PI4KIIIβ inhibitor, BQR695, also exhibited antiviral activity against those coronaviruses. In conclusion, PKD and its substrate, PI4KIIIβ, may serve as novel antiviral targets for human coronaviruses and warrant further investigation.

**IMPORTANCE:**

Human coronaviruses can lead to a range of clinical symptoms, from asymptomatic infection to severe illness and death, with a limited array of antiviral drugs available. Protein kinase D (PKD) is involved in various cellular processes, such as cell proliferation, apoptosis, and membrane fission of the Golgi apparatus. However, the specific role of PKD in the human coronavirus life cycle remains unclear. In this study, we found that PKD inhibitors effectively attenuated human coronavirus replication at the trans-Golgi network (TGN) stage in the viral life cycle. Furthermore, inhibiting PKD reduced PI4KIIIβ activation, thereby blocking viral replication in the host cells. Importantly, PI4KIIIβ inhibitors also blocked human coronavirus replication. Thus, PKD may represent a promising therapeutic target against both current circulating and future emerging coronaviruses.

## INTRODUCTION

The COVID-19 pandemic has resulted in more than 170 million infections and over 3.5 million deaths worldwide. It has devastated more than 200 countries and territories, making it one of the most dangerous infectious disease outbreaks in human history (1, 2). The three highly pathogenic human coronaviruses, namely severe acute respiratory syndrome coronavirus (SARS-CoV), Middle East respiratory syndrome coronavirus (MERS-CoV), and severe acute respiratory syndrome coronavirus 2 (SARS-CoV-2) (3), cause the severe respiratory syndrome. Moreover, four common human coronaviruses (hCoVs) circulate widely and typically cause self-limiting, mild, upper respiratory tract infections in humans: 229E and NL63 (alphacoronaviruses) and HKU1 and OC43 (betacoronaviruses) (4). Although effective vaccines have been approved and distributed, virus variants, particularly those with mutations on the viral membrane protein spike, can still cause infection and illness. Therefore, the development of broad-spectrum antivirals against both currently circulating CoVs and future emerging CoVs is just as important as the development of vaccines.

Coronaviruses are a group of enveloped, positive-sense single-stranded RNA viruses. After the fusion of the viral and host membranes, viral proteins are expressed to release viral genomic RNA (5). Like other positive-strand RNA viruses, including picornaviruses and noroviruses, replication of the coronavirus genome occurs in membranous replication organelles (6–10). The establishment of the replication complexes is known to involve rearrangements of cellular membranes (11). Advanced electron microscopy observations have shown that coronaviruses bud inside the endoplasmic reticulum (ER)–Golgi intermediate compartment, and then travel through the Golgi and lysosome to reach the membrane for egress, a final step of the coronavirus replication cycle. The biogenesis of these complexes may involve multiple cellular sources of the membrane, such as the ER, Golgi apparatus, lysosome, and plasma membrane (12). The replication organelles are thought to concentrate host and viral factors required for viral RNA synthesis (8, 13). Numerous studies have demonstrated that positive-strand RNA viruses require endoplasmic reticulum (ER) and Golgi membranes for genome replication. Remodeling of these membranes is critical for replicating the virus (14). Research on the coronavirus envelope (E) protein has indicated its association with the trans-Golgi network (TGN), implying that membrane protein and lipid remodeling play a crucial role in generating viral replication complexes (15). Several host proteins are involved in the coronavirus replication complex, including the phosphatidylinositol 4-kinase IIIβ (PI4KIIIβ) enzyme linked to the development of SARS coronavirus infections (16). Proteomics analyses have identified additional potential components of the replication complex (17). SARS-CoV-2’s two phenylalanines in an acidic tract (FFAT)-like motif specifically binds to vesicle-associated membrane protein (VAMP)-associated protein A (VAP-A) (18).

PKD is a serine/threonine kinase family comprising three isoforms: PKD1, PKD2, and PKD3 (19). It is involved in numerous biological processes including cell differentiation, motility, vesicle secretion, gene transcription, and cytokine production (20). PKD mediates the Golgi localization of CERT at Ser132 and OSBP Ser240 proteins through binding to pleckstrin homology (PH) domains, inhibiting their functions in integrating cholesterol and sphingomyelin metabolism (21, 22). Active PKD promotes the secretion of cargo proteins. Multiple upstream pathways, such as the PKC pathway activated by PKC to generate DAG through phospholipase C (PLC), apoptosis through caspase cleavage, and Gβγ subunits, can activate PKD (23). PKD potentially interacts with Arf1, PI4KIIIβ, 14-3-3γ, and C-terminal-binding protein in a complex that facilitates vesicle budding and fission from the Golgi membrane (22). Thus, the evidence for a critical role of PKD in regulation of Golgi membrane remodeling and fission suggests that PKD may be involved in coronavirus replication (24).

Previous studies suggested that PKD regulates human rhinovirus replication (10), influenza A virus infection (19), herpes simplex virus one egress (20), and hepatitis C virus secretion (22). However, where PKD regulates human coronavirus replication remains unknown. In this study, we investigated the potential role of PKD in the regulation of the human coronavirus replication. We demonstrated that PKD is critically involved in coronavirus replication, and inhibiting PKD restricts coronavirus replication through the PI4KIIIβ-dependent signaling pathway.

## RESULTS

### The infection of common human coronavirus in cultured cells augments expression of PKD

**Fig 1:**
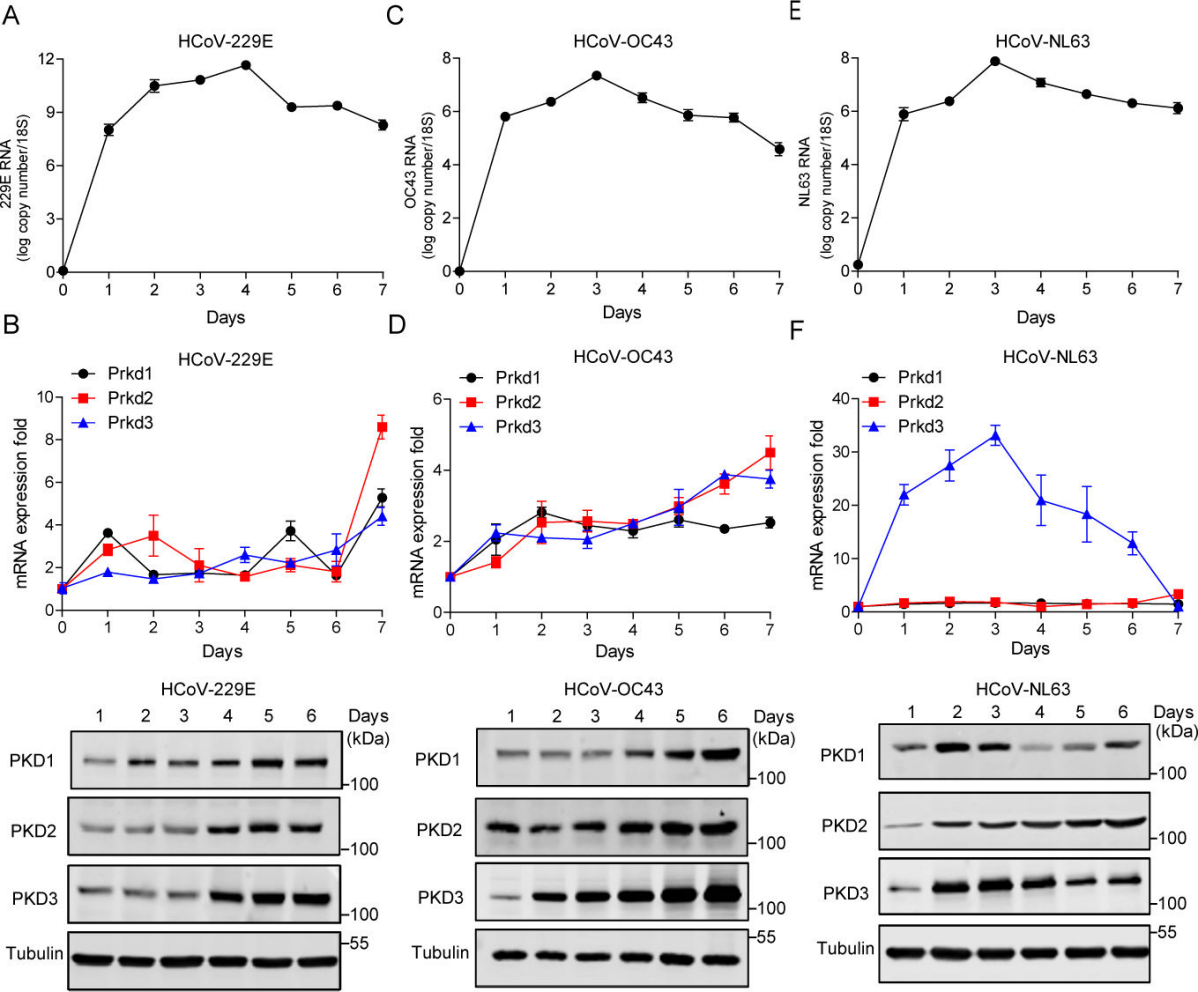
Infection by common human coronaviruses leads to an increased expression of PKD in cultured cells. (**A**) RT-qPCR was utilized to measure HCoV-229E viral RNA levels in MRC-5 cells, with results normalized to 18S RNA at various infection stages. (**B**) MRC-5 cells were infected with HCoV-229E at an MOI of 10 for a day, followed by 7 days of incubation period. Cell extracts were taken daily for RT-qPCR analysis (normalized to 18S RNA) and Western blotting (normalized to tubulin). Results are expressed as means ± SEM. (**C, D**) In a similar setup, MRC-5 cells were infected with HCoV-OC43 at an MOI of 10. (**E, F**) Another experiment was conducted using Caco-2-derived C2BBe1 cells infected with HcoV-NL63 at an MOI of 10. The results from these experiments represent data from three independent trials.

To ask where PKD is involved in HCoV replication, we infected Medical Research Council cell strain 5 (MRC-5) cells with human coronavirus HCoV-229E and HCoV-OC43, and then evaluated the expression of PKD in coronavirus-infected cells. The cells were infected with HCoV-229E or HCoV-OC43 at 20 multiplicity of infection (MOI) and incubated for 7 days post-infection (dpi). Then, the cells were harvested at different time points post-infection, and cell extracts were prepared for further analysis. Quantitative reverse transcription polymerase chain reaction (qPCR) and Western blot analysis of MRC-5 cell extracts from each time point were performed to detect *Prkd1*, *Prkd2*, and *Prkd3* mRNA and protein expression in coronavirus-infected MRC-5 cells. Consistent with previous studies, we confirmed that viral infectivity peaked at 4 and 3 days for HCoV-229E and HCoV-OC43, respectively (Fig. 1A and C). We found that both mRNA and protein levels of PKD1(*Prkd1*), PKD2 (*Prkd2*)*,* and PKD3 (*Prkd*3) were increased after HCoV-229E or HCoV-OC43 infection (Fig. 1A and B). Notably, *Prkd3* expression was significantly increased at 6 days post-infection. To further confirm PKD3 expression in HCoV, we repeated the study using the same strategy to test HCoV-NL63 on the C2BBe1 clone of the Caco-2 cell line (Fig. 1E and F). Both mRNA and protein levels of PKD3 (*Prkd*3) were significantly increased in HCoV-NL63-infected cells, whereas there were no significant changes of *Prkd1* and *Prkd2* mRNA levels. These results reveal that the PKD family members are implicated with HCoV infection, despite the differences in the expression of the three isoforms.

### PKD3 is essential for coronavirus replication

**Fig 2:**
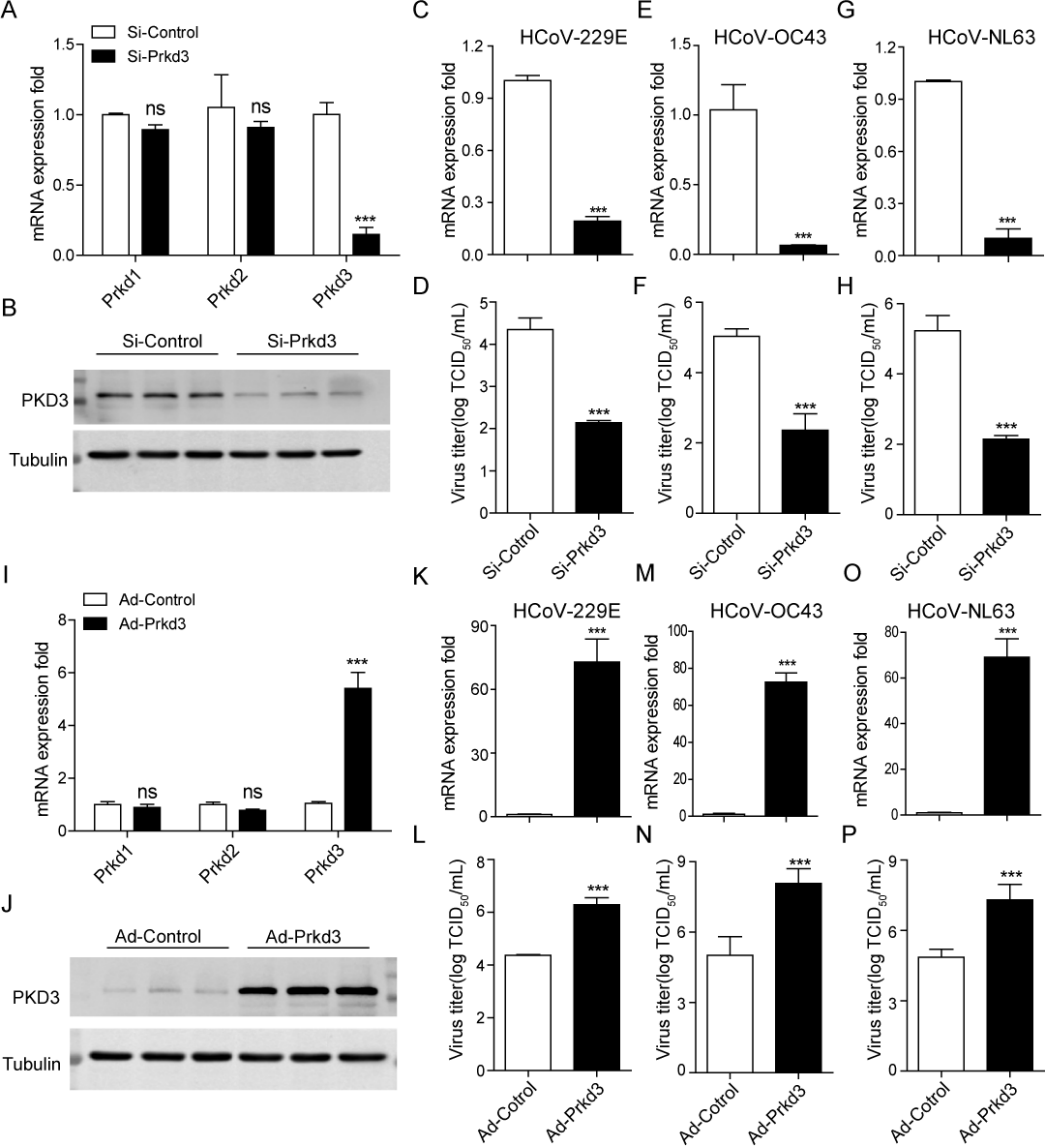
The inhibition of PKD3 attenuates common HCoV replication in cultured cells. (**A, B**) MRC-5 cells were transfected with PKD3 or control siRNAs for 48 h, after which RNA was extracted, and PKD levels were measured by RT-qPCR (normalized to GAPDH). Western blotting with antibodies for PKD3 and tubulin was also performed. (**C, D**) After infecting MRC-5 cells with HCoV-229E for 1 h, siRNAs were transfected for 48 h. The virus in the supernatants was quantified using RT-qPCR and plaque assays. (**E, F**) MRC-5 cells infected with HCoV-OC43 were analyzed similarly *via* RT-qPCR and plaque assays. (**G, H**) A similar experiment was performed in a C2BBe1 clone of Caco-2 cells infected with HCoV-NL63 and quantified by RT-qPCR and plaque assays. (**I, J**) The cells were infected with PKD3 adenovirus or control adenovirus for 48 h, after which RNA was extracted for PKD quantification by RT-qPCR (normalized to Gapdh), and Western blotting was performed. (**K, L**) After 1 h of HCoV-229E infection, MRC-5 cells were infected with PKD3 adenovirus or control adenovirus for 48 h, followed by viral quantification using RT-qPCR and plaque assays. (**M, N**) A similar process was applied for MRC-5 cells infected with HCoV-OC43. (**O, *P***) C2BBe1 cells infected with HCoV-NL63 underwent a similar experiment for viral quantification.

To investigate the involvement of PKD in HCoV infection, RNA interference was used to target one of the three PKD isoforms, PKD3, in a non-synchronized infection. The laboratory had previously used a small interfering RNA (siRNA) targeting *Prkd3* and a nontargeting siRNA (Si-Control) (25). RNAi was carried out for 48 h, during which PKD3 levels were reduced by approximately 80%, as measured by RT-qPCR (Fig. 2A) and Western blotting (Fig. 2B). MRC-5 cells were initially infected with HCoV-229E and HCoV-OC43 for 1 h, followed by 48 h of RNAi incubation, and the virus released into the extracellular medium was measured by plaque assays. The nontargeting Si-Control had no effect, but PKD3 interference significantly reduced HCoV-229E and HCoV-OC43 viral RNA expression and viral titers (Fig. 2C through F). Consistent with this, deficiency of PKD3 also substantially reduced HCoV-NL63 viral RNA expression and viral titers in the C2BBe1 clone of the Caco-2 cell line (Fig. 2G and H). In contrast, MRC-5 cells were overexpressed with PKD3 using *Prkd3* adenovirus and adenovirus LacZ as a control group. Both RT-qPCR and Western blotting confirmed that PKD3 was significantly overexpressed after a 24-h incubation (Fig. 2I and J). The HCoV-infected MRC-5 cells were overexpressed for an additional 48 h, and viral RNA levels were measured by RT-qPCR, showing a significant increase in both HCoV-229E and HCoV-OC43 viral RNA (Fig. 2K and M), as well as viral titers in the cell culture supernatants quantified by plaque assay (Fig. 2L and N). Moreover, viral RNA replication and viral titers of HCoV-NL63 were also significantly increased in PKD3-overexpressed C2BBe1 clones of Caco-2 cells (Fig. 2O and P). These data suggested that PKD3 specifically regulated HCoV replication.

### The PKD pharmacological inhibitor CRT0066101 attenuates HCoV replication in cultured cells

**Fig 3:**
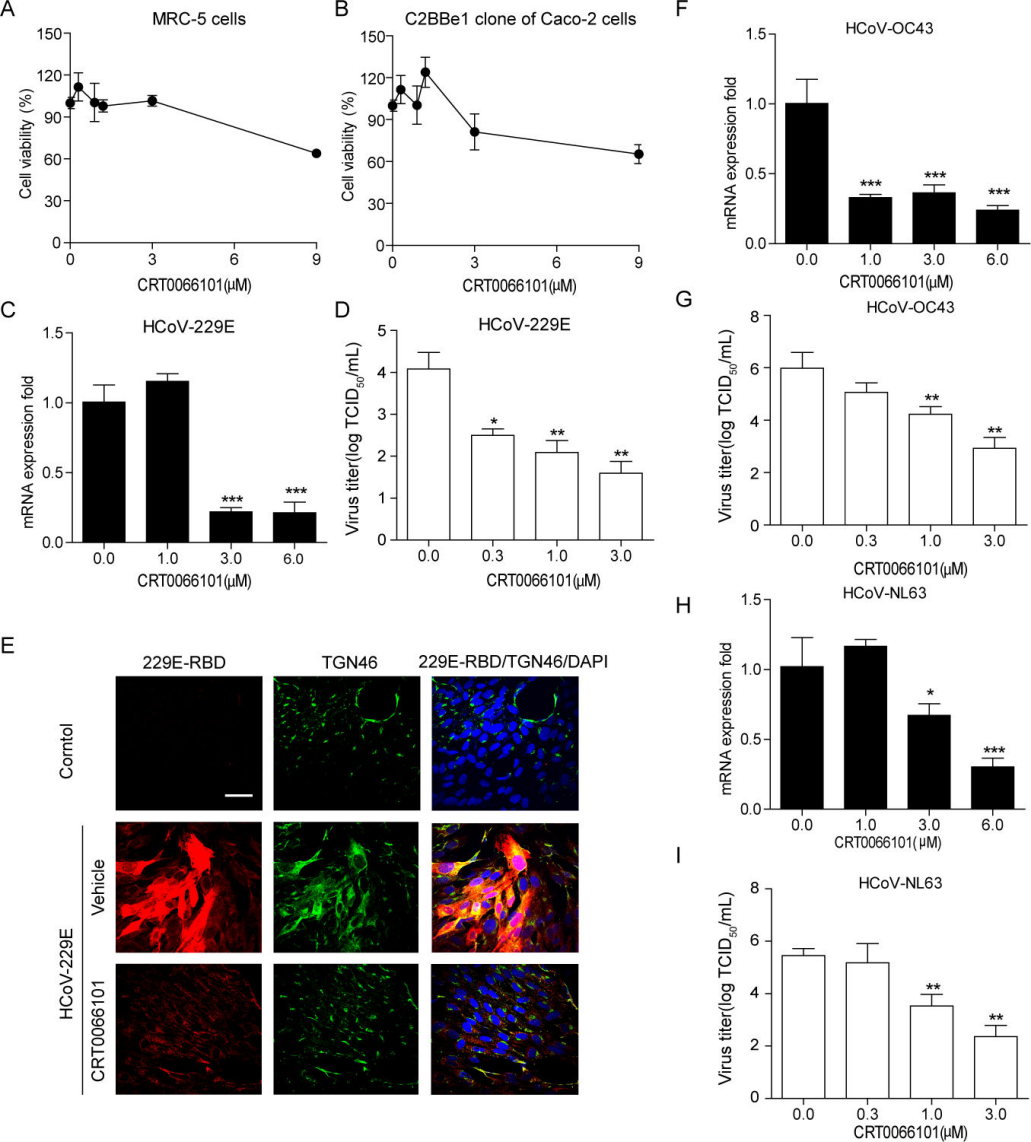
The PKD pharmacological inhibitor CRT0066101 reduces common HCoV replication. (**A, B**) MRC-5 and C2BBe1 cells were exposed to increasing CRT0066101 concentrations for 24 h, and cell viability was measured by CCK8 assays. (**C, D**) MRC-5 cells infected with HCoV-229E (MOI of 10) were treated with increasing CRT0066101 concentrations for 72 h. Viral replication was assessed *via* TCID50 endpoint titer, RT-qPCR (normalized to 18S RNA), and plaque assays. (E) MRC-5 cells infected with HCoV-229E were treated with CRT0066101, fixed, immunostained using anti-TGN46 and anti-229E-RBD antibodies, and analyzed *via* confocal microscopy (bar = 10 µm). (**F-H**) Cells infected with HCoV-OC43 or HCoV-NL63 (MOI of 10) were treated with CRT0066101, and viral replication was measured by TCID50, RT-qPCR, and plaque assays. Graphs represent the means (±SEM) from three independent experiments, with statistical significance evaluated using one-way ANOVA followed by Dunnett’s *post hoc* test. *, *P* < 0.05; **, *P* < 0.01; ***, *P* < 0.001.

We investigated the effect of chemical inhibitors of PKD on HCoV replication since PKD was found to be necessary for HCoV infection. CRT0066101, a PKD inhibitor, has been used in previous studies on HRV replication (10), HCV infection (22), and influenza A virus infection (19). To exclude the possible cell death involved in the inhibitory effect of CRT0066101, we used the CCK8 assay to measure the cell viability of MRC-5 cells and C2BBe1 clone of Caco-2 cells treated with CRT0066101 at different concentrations. Minimal cytotoxicity was only observed at the highest concentration tested, which was 9 µM. (Fig. 3A and B). We then quantified the effect of CRT0066101 on the replication of HCoV-229E, HCoV-OC43, and HCoV-NL63 in MRC-5 cells and C2BBe1 clone of Caco-2 cells by measuring viral titers through the determination of the 50% tissue culture infective dose (TCID_50_). Viral replication was subsequently measured by RT-qPCR at 3 dpi. As shown in Fig. 3C and D, CRT0066101 clearly inhibited viral mRNA replication and viral titers at concentrations above 3 µM. We investigated whether the Golgi apparatus was affected during coronavirus infection and whether CRT0066101 prevented HCoV-229E infection by infecting MRC-5 cells with HCoV-229E and treating them with CRT0066101 for 24 h. Immunofluorescence staining (Fig. 3E) revealed that the Golgi structure underwent fission in HCoV-229E-infected cells, and HCoV-229E was significantly decreased in PKD inhibitor-treated cells. We observed a dose-dependent inhibition of viral mRNA replication and viral titers with MRC-5 cells infected with HCoV-OC43, which showed that CRT0066101 inhibits both viral mRNA replication and viral titers at concentrations above 1 µM (Fig. 3F and G). We further tested CRT0066101 against HCoV-NL63 viral replication and demonstrated that it significantly reduced viral mRNA replication and viral titers in the C2BBe1 clone of Caco-2 cells at concentrations above 3 µM (Fig. 3H and I). Hence, we revealed that the PKD inhibitor CRT0066101 impeded common HCoV replication in cultured cells, and there was a suitable window of separation between efficacy and cytotoxicity.

### The PKD pharmacological inhibitor CRT0066101 reduces HCoV replication through impeding the fission of transport carriers from the trans-Golgi network

**Fig 4:**
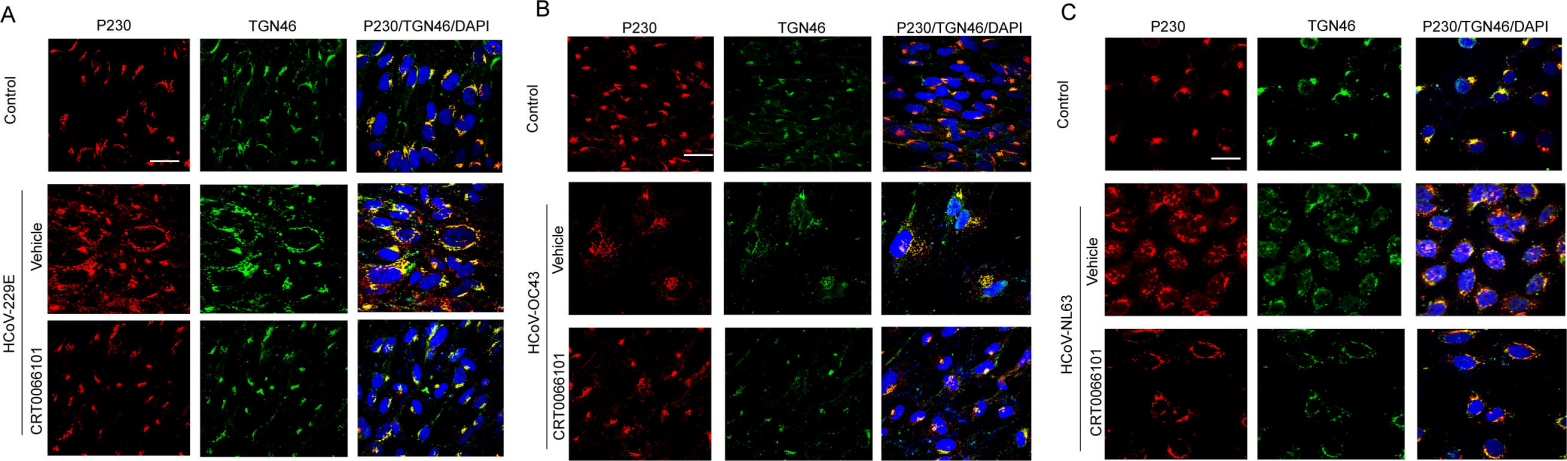
The PKD pharmacological inhibitor CRT0066101 blocks Golgi fission in the HCoV-infected cells. (**A**) MRC-5 cells infected with HcoV-229E and treated with CRT0066101 were immunostained using anti-TGN46 and anti-P230 antibodies, followed by confocal microscopy (bar = 10 µm). (**B, C**) The same process was repeated in MRC-5 and C2Bbe1 cells infected with HcoV-OC43 or HcoV-NL63 (bar = 10 µm).

It has been shown that the PKD inhibitor CRT0066101 does not inhibit viral entry or the clathrin-mediated endocytosis to affect HRV16 replication (10). Since PKD regulates TGN fission and vesicle formation and trafficking, it is likely that CRT0066101 may impede TGN fission, resulting in the blockage of HCoV replication. To test this possibility, we infected MRC-5 cells with HCoV-229E and HCoV-OC43 1 h before adding CRT0066101, and then examined the cellular Golgi architecture using immunofluorescence microscopy (Fig. 4A and B). In vehicle-treated cells with viral infection, we clearly observed Golgi membrane fission, while in CRT0066101-treated cells, Golgi membrane integrity appeared normal, and the Golgi morphology was consistent. CRT0066101-treated C2BBe1 clone of Caco-2 cells with HCoV-NL63 infection also displayed a normal Golgi membrane architecture compared with vehicle-treated cells with viral infection (Fig. 4C). Overall, these results suggest that the blockage of PKD-dependent Golgi fragmentation is likely to be involved in the inhibitory effect of CRT0066101 on HCoV replication.

### PKD is required for phosphatidylinositol 4-kinase iiiβ activation and promotes phosphatidylinositol 4,5-bisphosphate production

**Fig 5:**
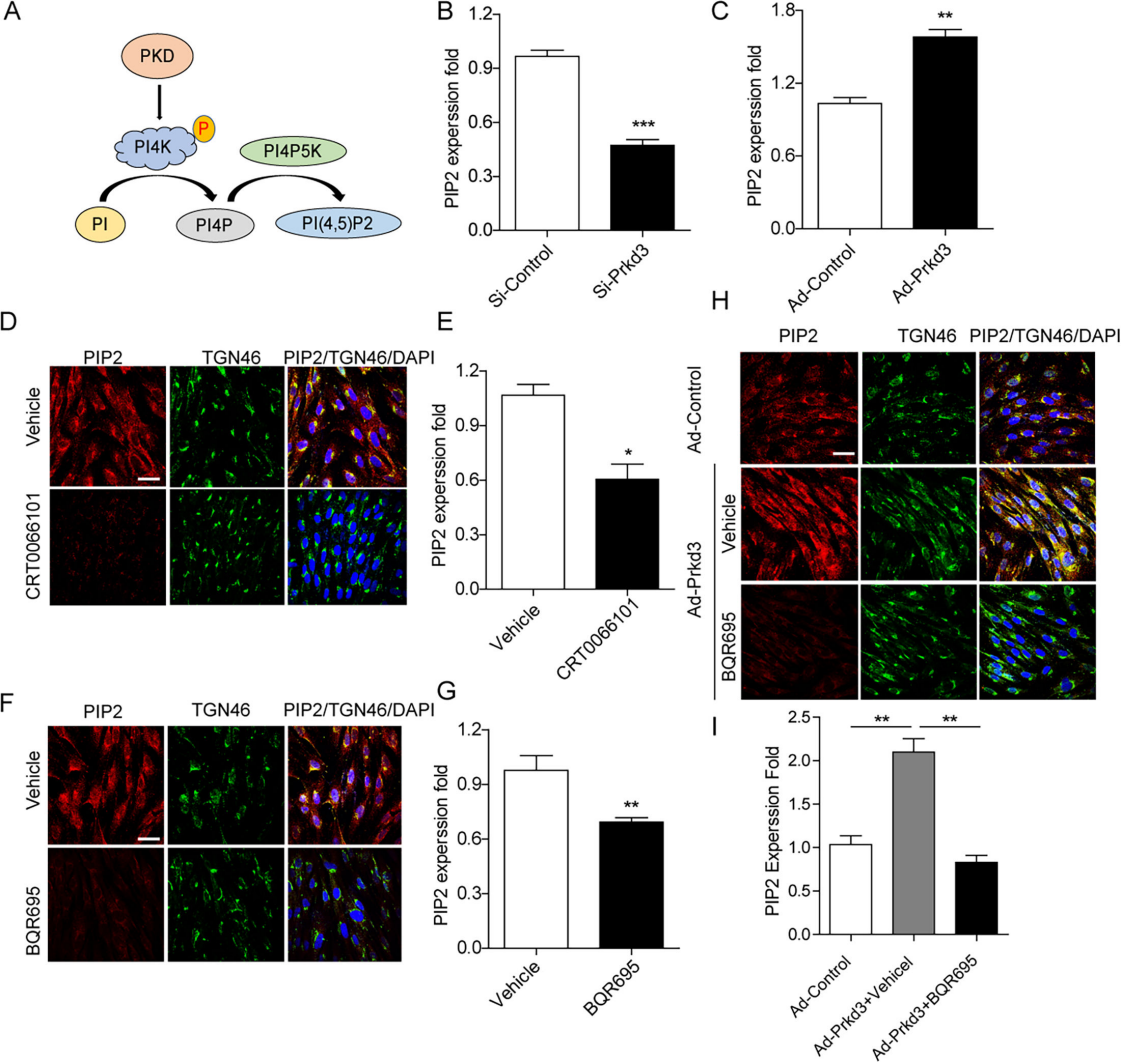
Inhibition of PKD decreases PIP2 production in cultured cells. (**A**) Model of the PKD-regulated PI4KIIIβ activation for PI kinases to PI4P phosphorylation followed by PI4P5K to induce PIP2 production. (**B**) MRC-5 cells were transfected with Si-Prkd3 or Si-Control for 24 h and then measured PIP2 expression by using live cell PIP2 assays. (**C**) MRC-5 cells were infected with Prkd3 adenovirus or adenovirus control for 24 h and then measured PIP2 expression by live cell PIP2 assays. (**D, E**) Cells-treated with CRT0066101 were immunostained with anti-TGN46 and anti-PIP2 antibodies and analyzed by confocal microscopy and live PIP2 assays. (**F, G**) MRC-5 cells treated with BQR695 were analyzed similarly. (**H, I**) PKD3-overexpressing MRC-5 cells were treated with BQR695 and quantified *via* confocal microscopy and PIP2 assays.

Previous research has shown that PKD controls numerous intracellular processes by phosphorylating various substrates. PKD can also phosphorylate PI4KIIIβ, which converts PI to PI4P (26). PI4P is essential for the generation of PI(4, 5)P2, which is phosphorylated by PI4P5K and is important for signaling (27) (Fig. 5A). It was postulated that inhibiting PKD could prevent antiviral activity by blocking PI(4, 5)P2 signaling. To test this hypothesis, we knocked down PKD3 in MRC-5 cells using siRNA targeting *Prkd3* and a nontargeting siRNA as a control, and then incubated the cells for 24 h. The cells were then transduced with sensor BacMam and sodium butyrate and allowed to rest at room temperature for 30 min before being incubated for an additional 24 h to measure fluorescence. We found that PI(4, 5)P2 expression was significantly decreased in PKD3-deficient MRC-5 cells compared with the control (Fig. 5B). To assess PI(4, 5)P2 expression in PKD3-overexpressing MRC-5 cells, the cells were infected with PKD3 adenovirus for 24 h and then transduced with sensor BacMam and sodium butyrate for an additional 24 h. The results demonstrated that PI(4, 5)P2 levels were increased in PKD3-overexpressing MRC-5 cells (Fig. 5C). We also tested whether CRT0066101, a PKD inhibitor, could block PI(4, 5)P2 expression using immunofluorescence microscopy and Green Fluorescent PIP2 Assay. The cells were treated with 3 µM CRT0066101 for 24 h, and we observed a significant reduction in PI(4, 5)P2 expression, which was confirmed by Green Fluorescent PIP2 Assay (Fig. 5D and E). BQR-695 is a PI4KIIIβ inhibitor with an IC50 of 80 nM for human PI4KIIIβ, respectively. To validate that PI4KIIIβ is involved in PI(4, 5)P2 production, MRC-5 cells were treated with 0.5 µM BQR-695 for 24 h to inhibit PI4KIIIβ activation. The cells were then examined using fluorescent microscopy and Green Fluorescent PIP2 Assay to detect PI(4, 5)P2 expression (28). We found that PI(4, 5)P2 expression in the cells was significantly decreased compared with the control cells (Fig. 5F and G). Furthermore, pre-incubating MRC-5 cells with PKD3 adenovirus for 24 h and then treating them with 0.5 µM BQR-695 for 24 h also decreased PI(4, 5)P2 expression in the cell membrane and Golgi apparatus (Fig. 5H and I). We conclude that the PKD inhibitor CRT0066101 blocks PI(4, 5)P2 expression by inhibiting PI4KIIIβ activation, indicating that a PI4KIIIβ inhibitor could also prevent viral replication.

### The PI4KIIIβ inhibitor BQR-695 inhibits HCoV replication through blocking the fission of transport carriers from the trans-Golgi network

**Fig 6:**
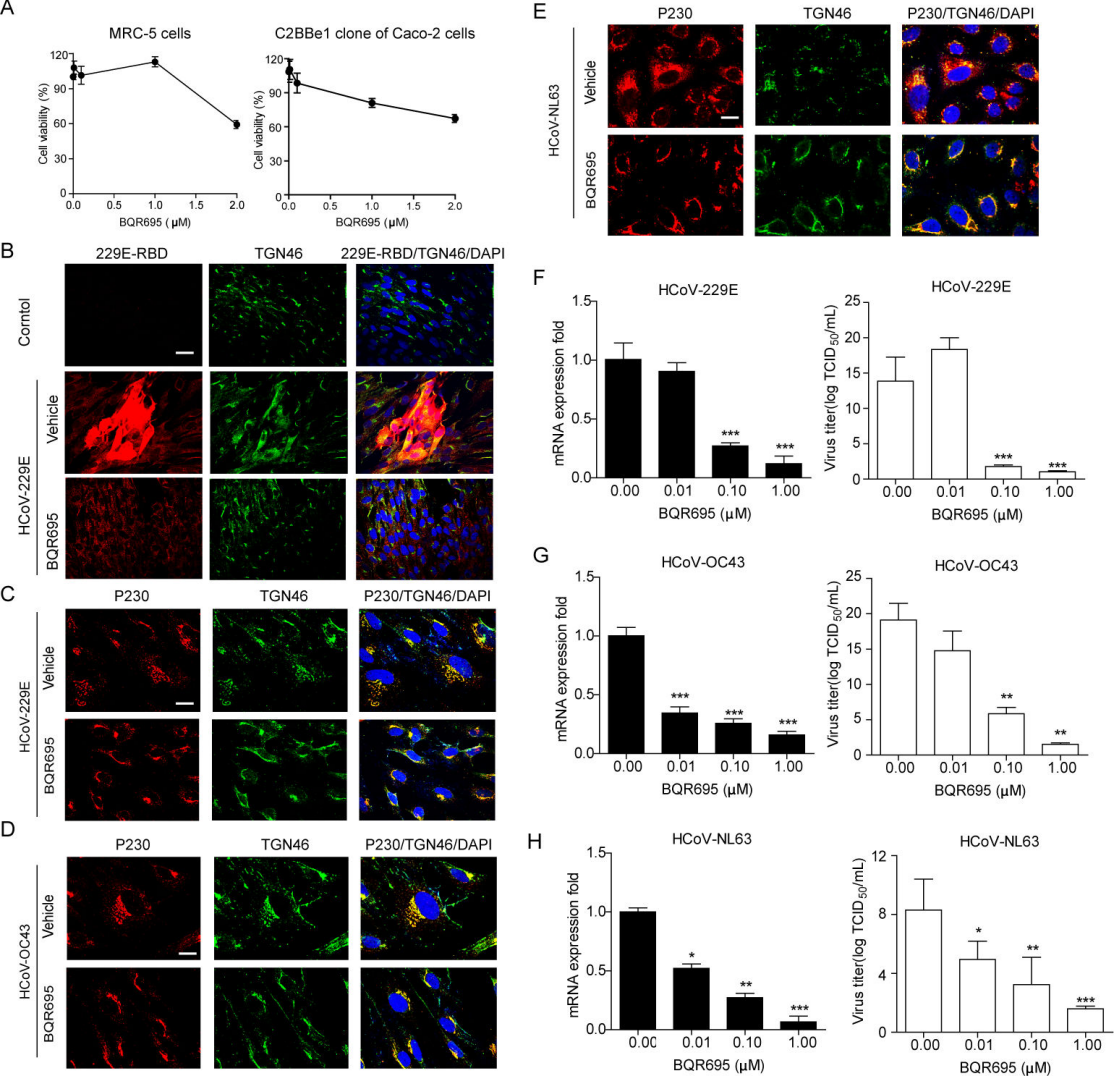
The PI4KIIIβ inhibitor BQR695 attenuates common HCoV replication and Golgi fission in cultured cells. (**A**) MRC-5 cells were treated with varying concentrations of BQR695 for 24 h, with cell viability measured by CCK8 assays. (**B, C**) HCoV-infected MRC-5 cells were treated with BQR695, fixed, and immunostained with anti-TGN46 and anti-P230 antibodies for confocal microscopy analysis (bar = 10 µm). (**D**) C2BBe1 clone of Caco-2 cells infected with HCoV-NL63 and then treated with BQR695, fixed, immunostained, and analyzed by confocal microscopy (bar = 10 µm). (**E-H**) MRC-5 and C2BBe1 cells infected with HCoV-229E, HCoV-OC43, or HCoV-NL63 were treated with BQR695, and viral replication was assessed by TCID50 and RT-qPCR. (**I-J**) Caco-2 cells, specifically the C2BBe1 clone, were infected with HCoV-NL63 at a multiplicity of infection (MOI) of 10. After allowing replication to proceed for 1 h, the cells were exposed to increasing concentrations of CRT0066101 for 24 h. *, *P* < 0.05; **, *P* < 0.01; ***, *P* < 0.001.

To investigate whether PI4KIIIβ is involved in the HCoV infection process through Golgi fission, we used a PI4KIIIβ inhibitor called BQR-695 to block its activity (28). Cell viability assays showed that BQR-695 had no cytotoxic effects on MRC5 and C2BBe1 Caco-2 cells at a concentration of ≤2.0 µM (Fig. 6A). We then infected MRC-5 cells with HCoV-229E and treated them with BQR695 for 24 h to confirm whether the Golgi apparatus was affected during coronavirus infection and whether BQR695 prevented HCoV-229E infection. Immunofluorescence staining (Fig. 6B) showed that Golgi structures were under fission in HCoV-229E-infected cells, and that HCoV-229E has significantly decreased in BQR695-treated cells. After excluding the cytotoxicity of BQR-695, MRC5 cells were infected with HCoV-229E and HCoV-OC43 at a MOI of 10 (50% tissue culture infective dose [TCID_50_]/cell) 1 h prior to BQR-695 addition, and then the cells were stained with TGN markers and detected using confocal microscopy (Fig. 6C and D). We observed that compared with the vehicle treatment, BQR-695 concentrations of 1 µM resulted in normal Golgi membrane staining, and the Golgi morphology was normal in the cell culture at 24 h. We used a similar strategy to treat C2BBe1 Caco-2 cells with HCoV-NL63 infection, followed by BQR-695 concentrations of 1 µM. The results also displayed a normal Golgi membrane architecture in BQR-695-treated cells compared with vehicle-treated cells with viral infection (Fig. 6E). Furthermore, after the MRC5 cells were infected with HCoV-229E and HCoV-OC43 at an MOI of 10 for 1 h, the cells were treated with different doses of BQR695 for 24 h. The viral RNA expression was significantly lower than the control group, and viral titers were also significantly reduced under 0.1 and 1 µM BQR-695 treatment (Fig. 6F and G). At 1 h after HCoV-NL63 infection, C2BBe1 Caco-2 cells were treated with dose-dependent BQR695. The viral RNA expression was reduced in drug-treated cells at 24 h compared with the vehicle treatment, respectively. The HCoV-NL63 titers were further determined by endpoint dilution assay and also showed the inhibitory effect of BQR695 (0.1 and 1 µM) on infectious HCoV-NL63 production (Fig. 6H). These results indicate that the activity of PI4KIIIβ is essential for common HCoV replication and suggest that PI4KIIIβ mediates HCoV replication by regulating the fission of cellular Golgi.

### Both CRT0066101 and BQR695 attenuate HCoV replication in Vero-E6 cells

**Fig 7:**
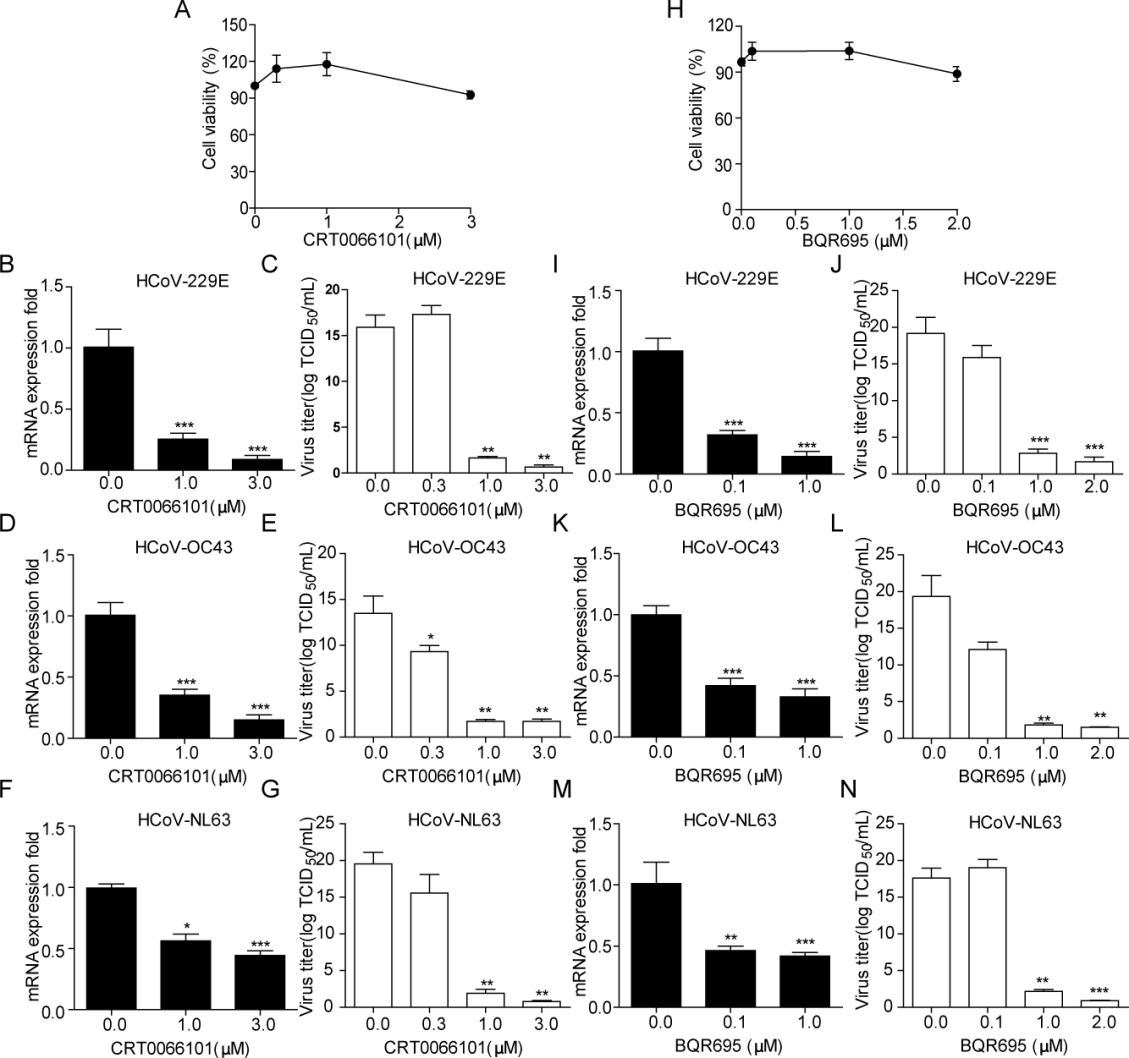
CRT0066101 and BQR695 are both effective in inhibiting HCoV replication in Vero-E6 cells. (**A**) Vero-E6 cells were treated with increasing concentrations of CRT0066101 for 24 h, and cell viability was assessed using a CCK8 assay. (**B-G**) The cells were infected with HCoV-229E, HCoV-OC43, or HCoV-NL63 at an MOI of 10. After a 1-h infection period, the cells were treated with increasing concentrations of CRT0066101 for 24 h. Viral replication was assessed using TCID_50_, and viral RNA levels in the supernatants were quantified by RT-qPCR, with normalization to 18S RNA. (**H**) Vero-E6 cells were also treated with increasing concentrations of BQR695 for 24 h, with cell viability determined by CCK8 assay. (**I-N**) Following infection with HCoV-229E, HCoV-OC43, or HCoV-NL63 at an MOI of 10 and a 1-h infection period, the cells were exposed to escalating concentrations of BQR695 for 24 h. Viral replication was measured by TCID50, RNA was extracted from the supernatants, and viral RNA levels were quantified using RT-qPCR, normalized to 18S RNA.

The critical role of CRT0066101 and BQR695 in modulating Golgi structures and their effect as inhibitors in HCoV-infected MRC-5 cells led us to explore the potential antiviral effects of CRT0066101 and BQR695 on other cells. Initially, we confirmed that CRT0066101 did not exhibit cytotoxic effects in Vero-E6 cells at varying concentrations. Our findings indicate that cell viability was unaffected by concentrations of CRT0066101 up to 3 µM (Fig. 7A). We then evaluated the effect of CRT0066101 on Vero-E6 cells infected with HCoV-229E for 1 h, allowing the virus to egress the cells. Treatment with CRT0066101 resulted in a dose-dependent reduction of viral replication and viral titers, with a significant decrease observed at 3 µM (Fig. 7B and C). Furthermore, HCoV-OC43 and HCoV-NL63 replication was also found to be reduced in the presence of 3 µM CRT0066101 in Vero-E6 cells (Fig. 7D–G), indicating that the observed antiviral effect of CRT0066101 is not cell type-specific. To exclude the possibility of cytotoxicity caused by BQR695 in Vero-E6 cells, we conducted a CCK-8 assay, demonstrating that BQR695 did not have an obvious impact on cell viability (Fig. 7H). Consistent with the CRT0066101 data, BQR695 was confirmed to reduce HCoV-229E replication at non-toxic concentrations (Fig. 7I and J). Additionally, the inhibitor of PI4KIIIβ also exhibited similar antiviral effects on HCoV-OC43 and HCoV-NL63 replications in Vero-E6 cells (Fig. 7K–N). Collectively, these data support a crucial role of PKD and PI4KIIIβ in common HCoV replication, indicating that CRT0066101 and BQR695 represent promising antiviral drug targets for broad-spectrum antivirals against coronaviruses without cell-type specificity.

## DISCUSSION

In this study, we first demonstrated the involvement of protein kinase D (PKD) in the replication of common human coronaviruses through the activation of PI4KIIIβ. Our subsequent investigation showed that PKD3 positively regulates HCoV replication in human MRC-5 cells. Interestingly, our data revealed that PKD promotes HCoV replication by inducing Golgi fission. Moreover, PKD stimulates PI4KIIIβ activation at the trans-Golgi network to regulate TGN fission and vesicle trafficking. To further explore the role of PKD in HCoV replication, we treated infected cells with the PKD inhibitor, CRT0066101, which significantly reduced viral replication. This result suggests that PKD is an important target for antiviral replication. Additionally, we demonstrated that PI4KIIIβ is regulated by PKD and is also involved in viral replication. PI4KIIIβ inhibitors exhibited significant antiviral effects against infectious coronaviruses, including HCoV-OC43, HCoV-NL63, and HCoV-229E. In summary, our results demonstrate that PKD and PKD-mediated PI4KIIIβ activation are key factors contributing to coronavirus replication. These findings provide compelling evidence to support the inhibition of PKD and PI4KIIIβ activation as broad-spectrum antivirals for coronaviruses.

Previous studies have shown that PKD regulates Golgi membrane fission and fusion, TGN-to-plasma membrane cargo transport, and lipid homeostasis (20, 29, 30). However, it was largely unknown whether the PKD pathway is implicated in human coronavirus replication. To investigate this, we first examined whether common HCoV infection activates PKD by analyzing cell lysates from virally infected MRC5 cells and C2BBe1 clones of Caco-2 cells. Using RT-qPCR, we observed a significant increase in the expression of all three PKD family proteins (*Prkd1*, *Prkd2*, and *Prkd3*) in human cells infected with HCoV. Notably, PKD3 expression was observed to be in contrast to PKD1 and PKD2 from the early to late stages of infection, indicating the potential for different PKD family proteins to play distinct roles in the replication cycle. Knockdown of PKD3 through RNA interference led to inhibited viral RNA replication and viral titers in HCoV infection, while overexpression of PKD3 through adenovirus significantly promoted viral replication, suggesting the involvement of PKD in coronavirus replication.

To investigate the essentiality of PKD activation for the viral life cycle, we evaluated the effectiveness of CRT0066101, a widely used PKD inhibitor that has previously demonstrated antiviral activity against various viruses, including human rhinovirus replication (10), influenza A virus infection (19), herpes simplex virus one egress (20), and hepatitis C virus secretion (22). Animal models have also shown that CRT0066101 is well-tolerated at doses up to 80 mg/kg of body weight (31). Our results demonstrated that CRT0066101 at a concentration of 3 µM completely inhibited RNA replication and viral titers in HCoV-OC43- or HCoV-NL63-infected MRC-5 cells and HCoV-229E-infected C2BBe1 clone of Caco-2 cells. Notably, this concentration was below the cytotoxic dose, as demonstrated by cell viability assays. Therefore, our findings suggest that CRT0066101 effectively prevents viral RNA replication and release, which are crucial late events in the viral replication cycle.

In this study, we investigated the potential mechanisms by which PKD inhibitors block coronavirus replication. One possibility is that PKD influences PI4K activation, as previously proposed (26). Studies have shown that PI4KIIIβ, a key player in Golgi complex structure, is a physiological substrate of PKD (26, 32). PKD phosphorylates PI4KIIIβ to stimulate lipid kinase activity and enhance vesicle transportation (33). PI4KIIIβ converts phosphoinositide (PI) to phosphatidylinositol 4-phosphate (PI4P), which mediates Golgi membrane localization (26). PI4P is phosphorylated to phosphatidylinositol 4,5-bisphosphate PI(4, 5)P2 by phosphatidylinositol 5-kinase activation (32). We indirectly examined the activation of PI4KIIIβ by examining PI(4, 5)P2 production using a PIP2 assay, as we could not obtain the PI4KIIIβ phosphorylation antibody to directly reflect the phosphorylation of PI4KIIIβ. We confirmed that PKD mediates PI4KIIIβ activation, as PI(4, 5)P2 production was significantly decreased in MRC-5 cells treated with PKD inhibitor. This finding adds significant confidence to the previous observation that PI4KIIIβ activation is regulated by PKD (26).

The importance of PI4KIIIβ in regulation of viral entry and replication has been well documented. For examples, several studies have shown that PI4KIIIβ is essential for picornavirus RNA replication (17, 34–36). A recent study has also indicated that PI4KIIIβ is implicated in SARS-CoV entry to host cells (37). Mechanistically, it has been shown that PI4KIIIβ is crucial for controlling Golgi membrane dynamics and vesicular sorting events (38). Our results showed that inhibition of PI4KIIIβ greatly decreased the replication and viral titer of common human coronaviruses by blocking TGN fission and vesicle trafficking. These data suggest that the lipid cycle between the cell membrane and TGN significantly affects coronavirus entry and replication. In agreement with our results, a recent study showed that the PI4KIIIβ inhibitor, bithiazole, blocks the replication of SARS-CoV-2 at low micromolar and sub-micromolar concentrations (39). However, the underlying mechanism has yet to be confirmed. In this study, we confirmed that BQR695, a PI4KIIIβ inhibitor, also reduces coronavirus replication in HCoV, including HCoV-OC43, HCoV-NL63, and HCoV-229E. Therefore, BQR695 should be further investigated for its potential to inhibit SARS-CoV-2 replication.

**Fig 8:**
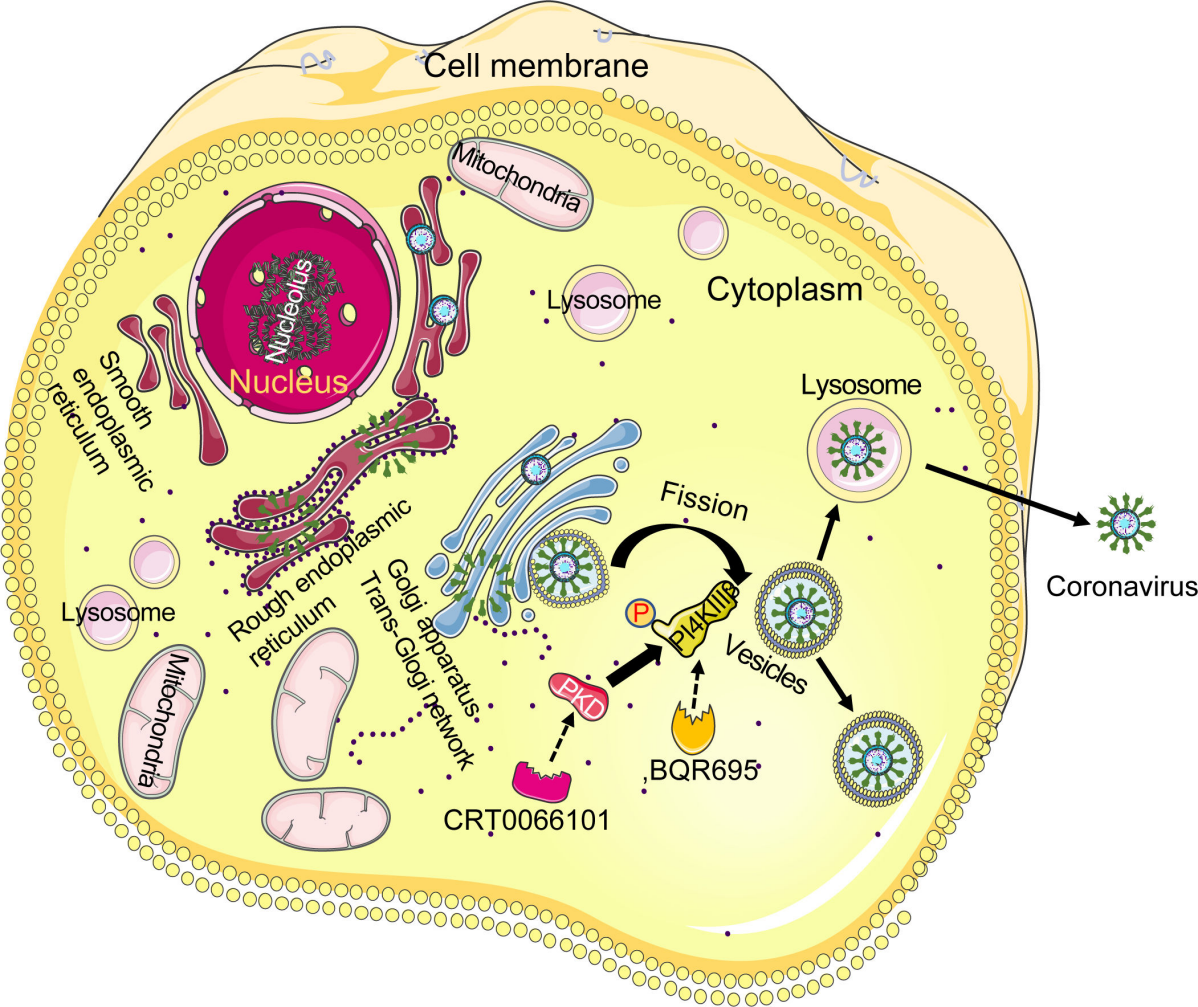
A working model for the role of PKD in the regulation of coronavirus replication in host cells. PKD-mediated phosphorylation of its substrate PI4KIIIβ enhances the fission of vesicles from the trans-Golgi network in coronavirus-infected cells by stimulating the lipid kinase activity of PI4KIIIβ. Inhibition of PKD or PI4KIIIβ prevents coronavirus replication by blocking Golgi fission.

In this study, we propose a model (see Fig. 8) for a potential role of PKD in coronavirus replication cycle. We suggest that PKD-mediated phosphorylation enhances the fission of vesicles from the TGN in coronavirus-infected cells by stimulating the lipid kinase activity of PI4KIIIβ. Inhibition of PKD or PI4KIIIβ prevents coronavirus replication by blocking Golgi fission. Of note, several studies demonstrated that coronaviruses, including SARS-Cov2 and MHV and OC43, use lysosomal exocytosis for egress (40–43), a critical and final step of coronavirus replication cycle. While this study did not specially address whether the PKD/PI4PIIIβ pathway regulates coronavirus egress from the host cells, it is likely that the PKD/PI4PIIIβ-dependent GNT alternation would also affect coronavirus egress through lysosomal exocytosis, since lysosomes are formed by the transport vesicles budded from the TGN, and lysosome enzymes are made by proteins from ER and enclosed within vesicles by the Golgi apparatus. Further studies are necessary to determine whether the PKD/PI4PIIIβ pathway regulates virus entry to the cells, whether other pathways control coronavirus replication, whether the PKD/PI4PIIIβ signaling pathway regulates virus egress from the cells, and whether the PKD/PI4PIIIβ pathway regulates SARS-CoV-2 replication.

In summary, our study has identified PKD as a novel host factor that plays a crucial role in coronavirus replication by mediating PI4KIIIβ activation. Our findings not only enhance the mechanistic understanding of coronavirus replication regulated by the PKD/PI4KIIIβ pathway but also suggest that the pharmacological inhibitors targeting the PKD/PI4KIIIβ pathway could be potential therapeutic drugs to combat coronavirus infections.

## MATERIALS AND METHODS

### Reagents

The Western blotting procedures utilized the following primary antibodies: rabbit anti-PKD3/PKCν (D57E6) Rabbit mAb (Cell Signaling; #5655S) and mouse anti-alpha tubulin antibody (Proteintech). Goat anti-rabbit/mouse IgG (H + L)-HRP conjugate was obtained from Bio-Rad and was revealed by using the Clarity™ Western ECL Substrate (Bio-Rad). For confocal microscopy, the primary antibodies included mouse PIP2 (PIP2 2C11) from Santa Cruz, rabbit Anti-TGN46 antibody from Abcam, HCoV-229E human coronavirus spike RBD antibody from R&D, and mouse anti-P230 from Fisher Scientific. Secondary antibodies included a donkey anti-rabbit conjugated to Alexa 546 (Invitrogen) and a donkey anti-mouse conjugated to Alexa 488 (Invitrogen).

### Cells and viruses

Medical Research Council cell strain 5 (MRC-5) cells and Vero-E6 (African green monkey kidney) cells were obtained from Dr. Stephen Dewhurst at the Department of Microbiology and Immunology, University of Rochester Medical Center, Rochester, New York. C2BBe1 (clone of Caco-2) (human large intestine epithelial) (catalog no. CRL-2102) cells were purchased from ATCC. MRC-5 cells and Vero-E6 were grown in Dulbecco’s modified Eagle’s medium (DMEM; Sigma-Aldrich) supplemented with 10% bovine growth serum (HyClone) at 37°C with 5% CO_2_. C2BBe1 cells were also grown in DMEM supplemented with 10% bovine growth serum and 0.01 mg/mL human transferrin.

Human coronaviruses, including HCoV-OC43, HCoV-NL63, and HCoV-229E, were also provided by Dr. Stephen Dewhurst. HCoV-OC43 and HCoV-229E were propagated in MRC-5 cells and titrated using the same cell line to determine the TCID50/mL. HCoV-NL63 was grown in C2BBe1 cells and titrated in the same cell type. In HCoV infection experiments, cells were infected with either HCoV-OC43 or HCoV-229E at specific MOIs and time points. The infections were synchronized by incubating the virus for 1 h before transferring the cells to 37°C. HCoV-NL63 infections followed the same protocol.

### RNAi transfections

According to the manufacturer’s protocol, MRC-5 cells or C2BBe1 cells were transfected for 24 h with the transfection reagent only an siRNA targeting the human protein PKD3 (siPKD3) (25). As a negative nontargeting control, a scrambled version of Si-control was used. Transfections were performed using Lipofectamine 2000 (Invitrogen, Carlsbad, CA) at a 3:1 SiRNA in Opti-MEM serum-free medium (Invitrogen). The cells pre-infected with coronavirus at 10 MOI for 24 h and then transfected with siRNA to knockdown PKD3. Cell fractions and supernatants were harvested for assays.

### Prkd3 overexpression

The adenoviral vector was a serotype 5 adenovirus encoding *Prkd3*. MRC-5 cells or C2BBe1 cells were transfected for 24 h with the control adenovirus or *Prkd3* adenovirus (Vector Biolabs, ADV-219874) at 10 MOI in serum-free DMEM for 24 h in a growth medium. The cells were then directly harvested following this transfection or when indicated in the figure legends evaluated as described above.

### The treatment of the PKD inhibitor or the PI4KIIIβ inhibitor

CRT0066101, with a purity of 99.9%, was obtained from Tocris (44), while BQR695 was acquired from MedKoo (45). To study the effect of the inhibitors on viral replication, the HCoV was added to cells at MOIs ranging from 10 to 20. The cells were incubated at room temperature for 1 h, followed by PBS washes to remove unbound virus, and then treated with increasing doses of PKD inhibitors or an H_2_O vehicle control for 24 h at 37°C. After infection, cells and supernatants were collected and centrifuged at 10,000×*g* for 5 min at 4°C to eliminate debris. The supernatants containing the virus were used for TCID50 titrations. Cellular and viral RNA were extracted at the end of infection using RLT buffer, as detailed below.

### Live cell PIP2 assays

According to the manufacturer’s protocol, cells were detached and resuspended in complete culture media. Next, 20 µL of the sensor BacMam stock was mixed with 0.6 µL of the 500 mM SB (stock solution of sodium butyrate) and 24.4 µL of the complete culture media to prepare the transduction solution. Mix the cells with the transduction mix and seed them into each well of a 96-well plate. Incubate for 30 minutes at room temperature, then transfer the plate to the incubator for 24 hours. After incubation, measure the fluorescence intensity using an automated fluorescence plate reader.

### Cell viability assays

Cell viability assays were conducted in parallel using uninfected MRC-5, C2BBe1, and Vero-E6 cells treated with either CRT0066101 or BQR695. Three hours before the end of treatment, CCK8 solution was added per the manufacturer’s protocol. After incubation at 37°C for an additional 4 h, cell viability was analyzed using a microplate reader.

### Western blotting

Following either siRNA transfection or adenovirus infection, cells were lysed in ice-cold lysis buffer with protease inhibitors (Sigma-Aldrich) and boiled for 10 min with 5× loading buffer. The cell lysates were loaded onto 10% Bis-Tris SDS-PAGE gels, followed by transfer to polyvinylidene difluoride membranes. Membranes were blocked in 5% bovine serum albumin for 1 h at room temperature. Primary antibodies were incubated overnight at 4°C, and secondary antibodies were incubated for 1.5 h at room temperature, and then followed by the addition of ECL reagent and data collection on an imgaeDoc (Bio-Rad). Analysis of quantified images was performed using ImageJ.

### Viral endpoint titer determination (TCID_50_)

For viral endpoint titers (TCID50), MRC-5, C2BBe1, or Vero-E6 cells were cultured in 96-well plates with DMEM containing 2% FBS. Virus was diluted eightfold in six replicates and incubated for 3 days. Titration was assessed by observing cytopathic effects (CPE) using the Karber method.

### Immunofluorescence microscopy

Immunofluorescence staining and microscopy were performed following previously described methods (46). Briefly, cells were fixed with 4% paraformaldehyde for 15 min at room temperature, permeabilized with 0.25% Triton X-100 for 15 min, and then blocked with 10% bovine serum albumin for 30 h. After incubating with primary antibodies overnight at 4°C, cells were stained with Alexa Fluor-conjugated secondary antibodies for 1.5 h and DAPI for DNA visualization. Fluorescence microscopy was carried out using an Olympus FV1000, and ImageJ was used for image analysis.

### Quantitative real-time PCR

Total RNA was extracted by lysing cells in RLT buffer with β-mercaptoethanol. mRNA was isolated using the RNeasy mini kit from Qiagen. For cDNA synthesis, 1 µg of mRNA was reverse-transcribed using the Omniscript RT kit at 37°C for 2.5 h. qPCR was performed using Bio-Rad’s CFX-96 system and SYBR Green Supermix, under the following conditions: 94°C for 10 min, 40 cycles of 94°C for 15 s, 58°C for 30 s, and 72°C for 30 s, followed by a final elongation at 72°C for 15 min. Quantification of the levels of mRNAs of interest was conducted by using primer, as follows: Human Prkd1 forward primer 5′- ACC CTT GGC TAC AGG ACT AT-3′, Human Prkd1 reverse primer 5′- CCA CCT CAG GTC ATC ACT TTC −3′ ; Human Prkd2 forward primer 5′- AGG GTT GGG TGG TTC ATT AC-3′, Human Prkd2 reverse primer 5′- TAT CTG TTG GTC GTG TTG TTC T-3′ ; Human Prkd3 forward primer 5′- TGT TCC CTG CAA CTG TTT CTG-3′, Human Prkd3 reverse primer 5′- CTC TCT CGT GTG AGG CCA ATC-3′ ; Human Gapdh forward primer 5′- GGT GAA GGT CGG TGT GAA CG-3′, Human Gapdh reverse primer 5′- CTC GCT CCT GGA AGA TGG TG-3′ ; HCoV 229E forward primer 5′- TTC CGA CGT GCT CGA ACT TT-3′, HCoV 229E reverse primer 5′- CCA ACA CGG TTG TGA CAG TGA-3′ ; HCoV OC43 forward primer 5′- ATG TTA GGC CGA TAA TTG AGG ACT AT-3′, HCoV OC43 reverse primer 5′- AAT GTA AAG ATG GCC GCG TAT T-3′ ; HCoV NL63 forward primer 5′- CTT CTG GTG ACG CTA GTA CAG CTT AT-3′, HCoV NL63 reverse primer 5′- AGA CGT CGT TGT AGA TCC CTA ACA T-3′ ; 18S rRNA forward primer 5′- CGC CGC TAG AGG TG A AAT TCT-3′, 18S rRNA reverse primer 5′- CAT TCT TGG CAA ATG CTT TCG-3′. Gene expression levels were normalized to 18S rRNA, and absolute quantification was performed using standard curves from DNA amplification.

### Statistical analysis

All data were expressed as the mean values ± standard deviations of the means (SEM). Statistical analyses were conducted using GraphPad Prism with Student’s *t*-test, one-way ANOVA, or two-way ANOVA when necessary, with a significance threshold of *P* < 0.05.

## Data Availability

All data supporting the conclusions of the paper are available in the article and corresponding figures.
